# PRMT5 function and targeting in cancer

**DOI:** 10.15698/cst2020.08.228

**Published:** 2020-07-13

**Authors:** Hyungsoo Kim, Ze'ev A. Ronai

**Affiliations:** 1 Cancer Center, Sanford Burnham Prebys Medical Discovery Institute, La Jolla, CA, 92037, USA.

**Keywords:** PRMT5, PRMT1, histone, transcription, splicing, MEP50, methylation, methyltransferase

## Abstract

Protein methyl transferases play critical roles in numerous regulatory pathways that underlie cancer development, progression and therapy-response. Here we discuss the function of PRMT5, a member of the nine-member PRMT family, in controlling oncogenic processes including tumor intrinsic, as well as extrinsic microenvironmental signaling pathways. We discuss PRMT5 effect on histone methylation and methylation of regulatory proteins including those involved in RNA splicing, cell cycle, cell death and metabolic signaling. In all, we highlight the importance of PRMT5 regulation and function in cancer, which provide the foundation for therapeutic modalities targeting PRMT5.

## INTRODUCTION

Posttranslational modifications (PTMs) are critical for proteome diversification. Protein modification on one or multiple sites can determine its conformation, subcellular localization, interaction with other proteins, stability, and/or activity. Such PTMs are mediated by diverse enzymatic processes, among them, phosphorylation, acetylation, ubiquitination, methylation and hydroxylation, and in turn reversed by enzymes that antagonize them, such as phosphatases, deubiquitinating enzymes, deacetylases and demethylases, to name a few.

Protein methylation on arginine residue was initially reported in late 1960s and early 1970s [1–3]. The first member of the protein arginine methyltransferase (PRMT) family, PRMT1 [4] was identified in 1996, followed soon after by nine others, including PRMT5. Initially identified as a 72-kDa pICln binding protein (IBP72, [5]) or JAK-binding protein 1 (JBP1, [6]), PRMT5 shares homology with the yeast proteins Skb1 (Shk1 kinase-binding protein 1 in *Schizosaccharomyces pombe*) and Hsl7p (histone synthetic lethal 7 in *Saccharomyces cerevisiae*). PRMT5 was later characterized as a distinct type of mammalian protein arginine N-methyltransferase [6–8].

Arginine-methylated proteins function in a number of key cellular processes required for maintenance of tissue homeostasis as well as diseases phenotypes. Use of both genetic (KO (knockout) mouse models) and pharmacological (small molecule inhibitors) tools has established the importance of arginine methylation in stem cell activity, development, neurodegenerative disease and cancer (reviewed in [9]). Among nine members of PRMT family, PRMT5, PRMT1 and CARM1 are most highly expressed in cancer (621 cancer cell lines from cancer cell line encyclopedia database) and such high expression is correlated with worse prognosis of patients in a number of cancer types. Accordingly, of 949 PRMT-related publications, most have focused on cancer, of which 35% studied PRMT5, 28% PRMT1, and 19% CARM1. These three PRMTs appear to promote oncogenesis through arginine-methylation-mediated control of gene expression, RNA splicing and DNA damage response (reviewed in [10]). These provide the foundation for clinical evaluation of PRMT5 (four clinical trials) and PRMT1 (one clinical trial) in cancer therapy. Here, we review current understanding of PRMT5, highlighting its role in cancer.

## PRMT5 STRUCTURE AND FUNCTION

PRMT family enzymes serve as “writers” of PTM, catalyzing three distinct types of methylation. In all, a methyl (-CH3) group from the methyl donor S-adenosylmethionine (SAM or AdoMet) is transferred to a guanidinium nitrogen of arginine on a target protein, generating a methylated guanidinium moiety and S-adenosylhomocysteine (SAH or AdoHcy), which is salvaged and re-used for methionine biosynthesis. In human cells, nine PRMTs catalyze three distinct methylation reactions. Type I PRMTs, including PRMT1, 2, 3, 6, 8, and CARM1 (PRMT4), catalyze ω-N^G^-monomethylarginine (MMA) and asymmetric ω-N^G,^ N^G^ – asymmetric dimethylarginine (aDMA). The type II PRMTs PRMT5 and 9 catalyze MMA and ω-N^G,^ N^G^ – symmetric dimethylarginine (sDMA). PRMT7, a type III PRMT, catalyzes only MMA. These modifications elicit a steric effect and change hydrogen bonding interaction of the methylated side chain, in turn altering molecular characteristics and function of the modified protein.

PRMT5 forms a unique hetero-octameric complex, which is composed of four PRMT5 proteins plus four essential cofactors, MEP50 (methylosome protein 50)/WDR77 (WD repeat domain 77). The unique N-terminal TIM barrel structure of the PRMT5 monomer enables formation of PRMT5 tetramer at the center of octameric complex and subsequent decoration of the PRMT5 tetramer with four MEP50 molecules [11, 12]. The PRMT5/MEP50 complex exhibits higher affinity to SAM and to the target substrate relative to a PRMT5 homodimer, resulting in a higher methylation activity of the hetero-octameric PRMT5/MEP50 complex [11].

The C-terminal catalytic domain of PRMT5 consists of two domains, the Rossam fold and β-barrel domains, required for binding cofactor (the methyl donor, SAM/AdoMet) and substrate, respectively. The structural restraints of substrate binding dictate preferential methylation of glycine-rich sequences, which allow conformational freedom of polypeptide chain to form a sharp β-turn [11]. Indeed, extensive proteomic analysis of immuno-enriched arginine methylation sites for PRMT5 indicates a preference for arginine flanked by glycines (at -1 and +1; e.g., GRG) rather than other amino acids within substrate peptides [13, 14].

## PRMT5 IN DEVELOPMENT, TISSUE HOMEOSTASIS AND CANCER

PRMT5 methylation of proteins is implicated in control of normal and pathological conditions, among them development, tissue homeostasis and cancer. Global PRMT5 KO in mice results in embryonic lethality [15], indicative of a developmental role. Conditional PRMT5 KO in specific tissues has allowed analysis of PRMT5 function in maintenance of tissue homeostasis, and survival and self-renewing capacity of stem/progenitor cells in nervous, muscular, hematopoietic and reproductive systems [9]. Here, we discuss PRMT5 function in the context of cancer-related activities.

### PRMT5 activity in tumor autonomous functions

PRMT5 plays a complex role in oncogenesis, as it is known to control expression of genes implicated in both tumor promotion and suppression (**Table 1**). The intrinsic roles of PRMT5 in cancer could be marked by altered expression of PRMT5 or of its adaptor proteins or by altered availability of factors that control its catalytic activity. Such deregulation of PRMT5 catalytic activity is often reflected by arginine methylation of target proteins functioning in either epigenetic regulation of gene expression, splicing, or signal transduction.

**TABLE 1.** Summary of PRMT5 substrates and the biological effect of their methylation by PRMT5.

**Substrate**		**Effect of methylation**	**Targets / Biological outcome**	Oncogenic/TS*	**References**	**Cancer type**
**Histones**	H3R8, H4R3	Transcription repression	ST7, NM23, RB1, RBL1, RBL2, CDH1	Oncogenic	[17–19, 29]	Leukemia, lymphoma, breast cancer
	H4R3	Transcription repression	Gas1, PTCH1, c-Myc	TS	[20, 21]	Pancreatic islet tumor
	H3R8, H4R3	Transcription repression	miRNAs targeting cyclinD1, c-Myc, FGFR3, FLT3	Oncogenic	[22–24]	Lymphoma, lung cancer, leukemia
	H3R8, H4R3	Transcription repression	Cul4A/B	Oncogenic	[67]	Lymphoma
	H3R8, H4R3	Transcription activation	eIF4E, FGFR3, AR	Oncogenic	[25, 26]	Colon cancer, prostate Cancer
	H3R2	Transcription activation	VIM, FOXP1, SLC7A11, RNF168	Oncogenic	[38–40, 88, 134]	Lung Cancer, breast cancer
	H4R3	Gene silencing	DNMT3A	ND	[31]	
**Transcription regulators**	TP53	Transcription activation	Transcription activation/cell cycle arrest, apoptosis	TS	[41, 42]	Sarcoma, lymphoma
	E2F1	Decrease protein half-life	Decrease protein half-life/promote growth or inhibit apoptosis	Oncogenic	[43]	Colon cancer
	RelA/p65	Enhance DNA binding	Enhance DNA binding/Activates NF-?B	Oncogenic	[45]	
	KLF4	Increase protein half-life	Increase p21/Cip1, repress BAX	Oncogenic	[48]	Breast cancer
	SREBP1	Increase protein half-life	Increase protein half-life/lipogenesis	Oncogenic	[49]	Hepatocellular carcinoma
	Androgen Receptor	Repress recruitment to target promoter	Repress recruitment to target promoter/repress AR target genes	Oncogenic	[51]	TMPRSS2:ERG positive prostate cancer
	SKI	Repress recruitment to target promoter	Repress recruitment to target promoter/Activates SKI target genes; SOX10, PAX3	Oncogenic	[53]	Melanoma
	BCL6	Enhance repressor activity	Enhance repressor activity/Repress BCL6 target genes	Oncogenic	[52]	Lymphoma
	FOXP3	Enhance activity	Enhance Treg function	Oncogenic	[103]	Regulatory T cell
**Splicing**	SmB/B', SmD1, SmD3	SnRNP assembly	Intact splicing		[59, 122, 123]	
	SRSF1	mRNA and protein binding	Intact splicing pattern	Oncogenic	[57]	AML
	ZNF326	Loss of activity in alternative splicing	Inclusion of A-T rich exons coupled mRNA decay	Oncogenic	[135]	Breast Cancer
**Signaling**	EGFR	Enhance EGFR-SHP1 interaction	Repress ERK activation	TS	[80]	Breast Cancer
	CRAF	Decrease protein half-life	Repress ERK activation	TS	[81]	Pheochromocytoma
	PDGFR	Increase protein half-life	Activates Akt/ERK signaling	Oncogenic	[82]	Oligodendrocyte
**DNA damage response**	RUVBL1	Activate TIP60	Enhance homologous recombination	ND	[91]	
	TDP1	Enhance activity	Repair DNA damage caused by Top1cc	ND	[92]	
	FEN1	Enhance recruitment to replication/repair foci	Intact replication and repair	ND	[89]	
	RAD9	Enhance activity	Intact cell cycle checkpoint	ND	[90]	
**Others**	PDCD4	Modify function	Enhance viability and growth	Oncogenic	[136, 137]	Breast Cancer
	IFI16	Inhibit activity	Repress STING activation	Oncogenic	[93]	Melanoma
	TRIM21	Inhibit E3 ligase activity	Inhibit IKKb degradation	Oncogenic	[84]	Multiple myeloma

* “TS*” and “ND” indicate “Tumor suppressive” and “not determined”, respectively.

### PRMT5 control of gene expression by histone methylation

**Figure 1:**
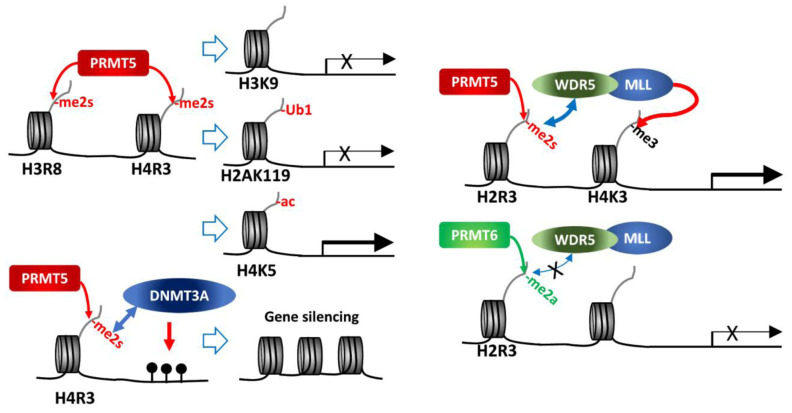
FIGURE 1: PRMT5 methylation of histone tails. PRMT5 methylation of histone tails results in activation/repression of target gene expression depending on subsequent modifications of histones or DNA.

Arginine methylation of histone tails is an epigenetic modification catalyzed by PRMT writers that controls gene expression. Type I or type II PRMTs introduce MMA, aDMA or sDMA to arginine residues in histone tails, enabling recruitment of specific reader(s) of those modified arginine residues, along with other chromatin remodeling modifiers. This in turn determines histone marks leading to gene activation or repression (**Figure 1**).

As a major type II PRMT, PRMT5 catalyzes methylation of four arginine residues within histone tails, namely, H4R3, H2AR3, H3R8 and H3R2 [6, 8]. Of those, H4R3me2s and H3R8me2s are generally associated with transcriptional repression. PRMT5 in association with components of the SWI/SNF chromatin remodeling complex (e.g., BRG-1, BRM, or BRD7) catalyzes histone methylations implicated in repression of the tumor suppressor genes ST7, NM23, RB1, RBL1 and RBL2 [16–19]. Direct interaction of PRMT5 with the MEN1 tumor suppressor in pancreatic islet tumors reportedly increases repressive sDMAs on H4 (H4R3me2s) and suppresses Gas1, PTCH1 and c-myc expression, thereby limiting oncogenic SHH (sonic hedgehog) signaling in these tumors. Notably, MEN1 mutations, which are frequently seen in inherited tumor syndromes, relieve repression by PRMT5, enabling oncogenic signaling and tumor growth [20, 21].

High PRMT5 expression in human cancers is implicated in tumor promotion through histone tail modifications that repress miRNAs that target tumor promoting genes. For example, in B cell lymphoma PRMT5 activity increases expression of cyclin D1 and c-myc in tumor cells by repressing miR-33b, miR-96 and miR-503. In lung cancer, PRMT5 activity increases FGFR3 expression by repressing miR-99, and in AML (acute myeloid leukemia) PRMT5 repression of miR-29b upregulates FLT3 [22–24].

While H3R8me2s and H4R3me2s are largely considered repressive marks, they are also implicated in transcriptional activation of some genes, as has been shown for FGFR3 and eIF4E expression in colorectal cancers [25], and AR (androgen receptor) expression in prostate cancers [26]. Indeed, PRMT5 depletion or inhibition downregulates roughly 50% of genes that exhibit 2-fold change (~1,300 genes) in AML, of which 53% (335 genes) restored expression upon inhibition of the H3K27 methyltransferase, EZH2. Further, H3K27me3 at the transcription start site of 25% of the 335 genes was reversed upon EZH2 inhibition [27]. These findings further highlight PRMT5-dependent regulation of gene expression. Notably, growing evidence support a crosstalk between different types of histone modification pointing to a complex, non-linear regulation. Accordingly, H3R8me2s results in deacetylation of H3K9 as a repressive mark [17], and H4R3me2s is associated with H4K5 acetylation as an active expression mark [28]. Thus, dynamic crosstalk between histone modifiers could serve as context-dependent histone marks influencing active/repressive gene expression.

H4R3 methylation by PRMT5 (H4R3me2s) was also linked with gene repression, through histone ubiquitination or DNA methylation. PRMT5 methylation of H4R3me2s results in its interaction with PHF1 (plant homeodomain (PHD) finger protein-1) through the N-terminal PHD domain, while its second PHD motif binds DDB1, a component of CRL4B-Ring E3 ligase complex. By bridging DDB1 to methylated histones PHF1 enables the monoubiquitination of H2AK119, which is essential to maintain PcG (polycomb group)-target gene repressive identity [29, 30]. PRMT5 methylation of H4R3 also recruits DNMT3A through the binding of H4R3me2s to the PHD motif in DNMT3A, thereby linking repressive H4R3me2s mark to DNA methylation that functions in gene silencing [31].

In contrast, PRMT5 methylation on H3R2 is generally associated with transcriptional activation of target genes. Notably, H3R2 can be methylated by PRMT5 or PRMT6, resulting in three types of methylation, H3R2me1, H3R2me2a or H3R2me2s. H3R2me2s, catalyzed by PRMT5, recruits WDR5 (a reader with a tudor domain recognizing sDMA) along with the MLL-family coactivator complex, resulting in H3K4me3, indicative of an active promoter, in the euchromatic loci [32–34]. On the other hand, H3R2me2a catalyzed by PRMT6 is enriched in heterochromatic or inactive euchromatic loci and is mutually exclusive with the presence of MLL-coactivator complex for H3K4me3 (**Figure 1**) [35–37]. Structure-based analysis of WDR5 binding to H3R2 harboring different types of arginine methylation reveals a binary switch: WDR5 binds equally to H3R2me0, me1 and me2s, but not to H3R2me2a [34]. Thus, PRMT5 methylated H3R2 (H3R2me1 or H3R2me2s) recruits WDR5/MLL complex resulting in gene activation. Along these lines, PRMT5 recruitment to the FOXP1 promoter facilitates FOXP1 expression via PRMT5-dependent H3R2me2s with concomitant WDR5/SET1/MLL complex-driven H3K4me3, implicated in maintenance of stemness in breast cancer stem cells (BCSCs) [38]. Genotoxic stress, including chemotherapy, induces interaction between β-catenin, ATM-phosphorylated JDP2 (Jun dimerization protein 2) and PRMT5, resulting in transcription of genes implicated in redox homeostasis. In this process, H3R2me1/H3R2me2s catalyzed by PRMT5 recruits the WDR5/MLL complex resulting in H3K4me3 and transcriptional activation of redox-related genes [39]. PRMT5 forms a complex with the adaptor protein SHARPIN implicated in H3R2me1 modification and recruitment of WDR5-ASH2 (MLL-component), facilitating formation of H3K4me3 on genes functioning in metastasis [40].

### PRMT5 control of gene expression by non-histone proteins

**Figure 2:**
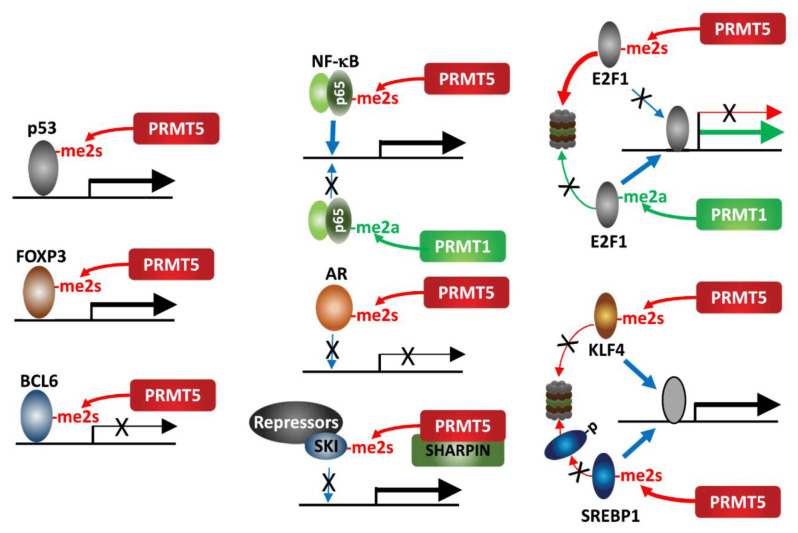
FIGURE 2: PRMT5 methylation of transcription factors. PRMT5 methylation of select transcription factors affects their activity, recruitment and stability.

Several non-histone proteins with tumor promoting or suppressing functions have been identified as PRMT5 substrates (**Figure 2**). STRAP, a factor recruited to the p53 complex during the DNA damage response (DDR), recruits PRMT5 and facilitates arginine methylation within the p53 oligomerization domain, decreasing p53 oligomerization which in turn increases nuclear retention of p53, with a concomitant increase in the expression of its target genes p21 and PUMA [41]. Likewise, PRMT5 methylation of p53 was implicated in altered nuclear localization and activity, which promotes lymphomagenesis [42].

An interesting example for the impact of methylation on protein function is provided by the opposing cellular functions of E2F-1, which are methylation-dependent. PRMT5 and PRMT1 methylate distinct arginine residues on E2F1 in a mutually exclusive manner, resulting in functionally opposing outcomes. The DDR induces E2F-1 methylation by PRMT1, which increases E2F-1 levels and transcriptionally activates genes implicated in promoting apoptosis. Conversely, PRMT5 methylation of E2F-1 is recognized by the tudor domain protein, p100-TSN, which decreases E2F-1 half-life and increases cell viability. During cell cycle progression, cyclin A binding to E2F-1 masks PRMT1 methylation of E2F-1, repressing its ability to promote apoptosis [43, 44].

PRMT5 methylation (sDMA) of arginine 30 in the DNA-binding domain of p65/RelA, a subunit of the NF-κB transcription factor, activates NF-κB transcriptional activity via enhancing its DNA binding affinity [45]. Conversely, PRMT1 methylation (aDMA) of the same residue represses DNA binding by p65/RelA following TNFα stimulation [46]. This switch highlights the importance of methylation type in defining NF-κB transcriptional outcomes. KLF4 (Kruppel-like factor 4), a zinc finger transcription factor, is methylated by PRMT5, which then blocks its ubiquitination by pVHL and stabilizes KLF4. Consequently, increased KLF4 availability contributes to breast cancer tumorigenesis by augmenting oncogenic signaling and expression of cell cycle genes [47, 48]. Likewise, PRMT5-methylation of SREBP1, a transcription factor required for *de novo* lipogenesis, prevents its phosphorylation by GSK3β and subsequent ubiquitination by FBXW7, thereby increasing lipogenesis and tumor growth of hepatocellular carcinoma (HCC) [49]. PRMT5 methylation of the TMPRSS2:ERG fusion protein is commonly seen in prostate cancer (PC) and implicated in prostate tumor formation by inhibiting AR -dependent transcription [50]. Mechanistically, PRMT5 interaction with the TMPRSS2:ERG fusion protein catalyzes methylation of arginine 761 in the AR LBD (ligand binding domain), which abrogates DNA binding, ligand-dependent AR activation and expression of AR-target genes [51]. PRMT5 methylation of R305 in BCL6, a transcriptional repressor and master regulator of normal GC (germinal center) formation and GC-derived B-cell lymphomagenesis, is required for its repressive activity on BCL6 target genes. Thus, PRMT5 inhibition derepresses BCL6 target genes, and suppresses DLBCL (diffuse large B-cell lymphoma) proliferation [52]. Our studies have identified SKI, a component of a transcriptional repressor complex antagonizing TGFβ signaling, as a substrate for PRMT5-MEP50-SHARPIN, which limits SKI recruitment to SOX10 and PAX3 promoters and derepresses them. In this context, PRMT5 control of SKI enhances melanoma growth by upregulating SOX10 and PAX3, which drive melanoma growth [53].

### Regulation of mRNA splicing

Growing evidence supports a crucial role of constitutive and alternative RNA splicing in control of genes driving cancer phenotypes [54, 55]. Thus, factors that genetically and epigenetically control activity of splicing machinery components must be tightly regulated to ensure splicing fidelity. Notably, protein arginine methylation is commonly seen on splicing machinery components. Moreover, proteome-wide profiling revealed enrichment of arginine-methylated proteins implicated in control of RNA splicing, transport or degradation [13, 14, 56, 57].

**Figure 3:**
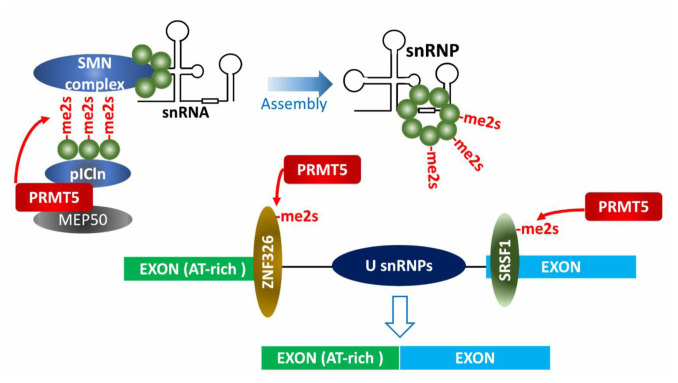
FIGURE 3: PRMT5 effect on splicing machinery. PRMT5 modification of splicing-machinery components is required for snRNP assembly and alternative splicing in pre-mRNAs, including those with a weak 5′ splice site (A-T rich), ensuring splicing fidelity.

Early studies identified PRMT5 as part of the 20S methylosome containing MEP50, pICln, SmD1, SmD3 and SmB, in which the three Sm proteins were methylated by PRMT5 (**Figure 3**) [5, 58, 59]. Methylated Sm proteins within the methylosome are then transferred to tudor domain containing SMN (survival of motor neuron) protein and assembled into snRNPs (small nuclear ribonucleoprotein particles) along with the 6S complex (pICln and Sm proteins) and snRNA. Assembled snRNPs in spliceosome then execute pre-mRNA splicing by recognizing sequence elements (e.g., 5′-, 3′- splice sites, branch point sequence and polypyrimidine tract) on pre-mRNA, in concert with other tans-acting splicing factors [8, 59, 60]. PRMT5 function in control of pre-mRNA splicing is conserved throughout evolution as it is detected in plants and flies [61–63].

In mouse neural progenitor cells, PRMT5 deletion causes defects in splicing of mRNAs with weak 5′ donor sites. Such defects take place in alternative splicing of Mdm4 in the absence of PRMT5, resulting in a shorter and less stable Mdm4 mRNA that activates the p53 pathway [64]. Indeed, PRMT5 knockdown or pharmacologic inhibition induces aberrant Mdm4 splicing enabling p53-mediated transcription of genes implicated in cell cycle and apoptosis, attenuating the growth of hematopoietic and solid tumors harboring wildtype p53 [64, 65]. Likewise, altered Mdm4 splicing and p53 activation, seen upon PRMT5 inhibition, overcomes melanoma resistance to CDK4/6 inhibition [66].

Notably, cyclinD1/CDK4 also induces MEP50 phosphorylation which increases PRMT5 activity [67], and correspondingly, melanoma that were treated with CDK4/6 inhibitor and developed resistance also exhibited high PRMT5 activity [65]. Whether the increased activity of PRMT5 contribute to the resistance and thus justifies possible combination of PRMT5 inhibitors and CDK4/6 inhibitors, remain to be determined.

PRMT5 control of splicing fidelity is also a factor in c-Myc-driven lymphomagenesis. Gene sets enriched in Eµ-myc B cell tumors include transcripts associated with snRNP biogenesis, RNA processing and RNA splicing, among them PRMT5. Myc- or PRMT5-depletion resulted in aberrant splicing (either exon skipping or retained introns) of genes associated with cell cycle arrest or apoptosis [68]. Consistently, large-scale proteomic profiling of PRMT5 substrates identified factors related to RNA processing [13, 57]. Among them, SRSF1, a serine/arginine rich protein functioning in alternative splicing, is directly methylated by PRMT5. Defects in PRMT5-methylation attenuate survival of AML cells by altering SRSF1 interaction with a subset of mRNAs and splicing-associated proteins (**Figure 3**).

TIP60/KAT5, a histone lysine acetyltransferase that drives homologous recombination (HR) DNA repair, is also regulated by PRMT5-mediated alternative splicing. In response to DNA damage, PRMT5 facilitates alternative splicing of TIP60/KAT pre-mRNA to a TIP60a isoform with higher H4 lysine acetylase activity, which ensures error-free HR DNA repair and maintenance of genome integrity in hematopoietic cells [69].

In addition to PRMT5 methylation that alters its transcriptional activity, E2F1 is also implicated in regulation of alternative splicing following PRMT5-methylation. E2F1 methylation promotes recruitment of p100/TSN as well as the snRNP spliceosome, which regulates splicing of E2F1 targets [70, 71]. Further studies are required to clarify mechanisms underlying the balance between E2F1 control of transcription and alternative splicing.

Given that numerous splicing-related proteins are methylated on arginine, it is not surprising that PRMT inhibition evokes defects in splicing fidelity of genes critical to cancer [13, 56, 57]. PRMT5 inhibition or depletion suppresses glioblastoma (GBM) growth by impairing removal of retained introns in genes functioning in cell proliferation, anti-senescence and anti-apoptosis [72]. Tumor intrinsic alteration of splicing through the frequently seen mutation in RNA splicing factors (SRSF2, SF3B1 and U2AF1) in AML cells confers vulnerability to either PRMT1 inhibition (MS023, [73]), PRMT5 inhibition (GSK3203591, [74]), or both [14]. Notably, combined treatment with type I PRMT inhibitors (GSK3368715 or MS023) and PRMT5 inhibitors (GSK3326595 or GSK3203591) slows *in vitro* and *in vivo* growth of tumor cells more effectively than treatment with either alone [14, 75]. Proteomic profiling of tumor cells revealed that either treatment alone reduces methylation of distinct subsets of protein, while combination treatment reduces the methylation in a higher number of proteins that serve as substrates for either type I, type II or both PRMTs. Consistently, transcriptomic splicing analysis confirms increase of aberrant splicing events following combination compared to single drug treatment [14, 75].

### PRMT5 regulation of translation

Tumor cells control protein translation as a means to adapt to tumor intrinsic and extrinsic stress conditions. PRMT5 reportedly interacts with and modifies (sDMA) RPS10 (ribosomal protein s10) which serves ribosome assembly. Such modification facilitates RPS10's ability to interact with NPM1 (nucleophosmin 1, a factor functioning in ribosome assembly), which ensures proper ribosome assembly, and general protein synthesis, supporting tumor cell proliferation [76]. hnRNPA1, an IRES transacting factor (ITAF) regulating IRES-dependent mRNA translation, is also methylated by PRMT5. Such methylation facilitates interaction of hnRNPA1 with an ITAD with concomitant translation of cyclin D1 and c-Myc [77]. Given the importance of IRES-dependent protein translation under stress conditions, PRMT5 control of IRES-dependent translation likely sustains oncogenic phenotypes under oxygen and nutrient deprivation, which are commonly seen in cancer.

### Control of growth factor signaling

In addition to transcriptional activation of FGFR genes by PRMT5 in lung and colon cancers [23, 25, 78, 79], direct arginine methylation by PRMT5 regulates activity of several proteins functioning in growth factor signaling pathways crucial to the proliferation, differentiation and survival of cancer cells.

**Figure 4:**
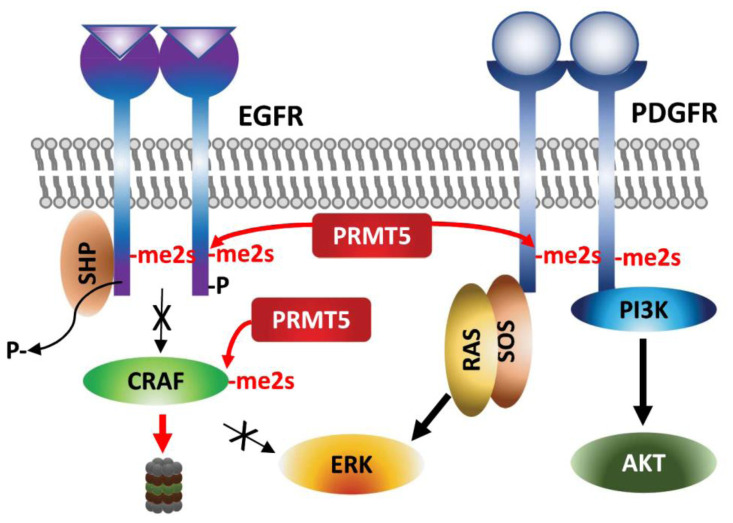
FIGURE 4: Regulation of growth factor receptors by PRMT5. Arginine methylations of the growth factor receptors EGFR and PDGFR affect ERK and AKT activation, respectively.

Arginine methylation of EGFR by PRMT5 occurs on a residue proximal to the auto-phosphorylated tyrosine following EGF-stimulation and thereby facilitates SHP recruitment and reduces ERK-activation and proliferation/migration of breast cancer cells [80]. Along the same line, genetic or pharmacologic (MTA, methylthioadenosine) inhibition of PRMT5 increases the amplitude of growth factor-driven RAS/ERK activation, by sustaining availability of activated CRAF by blocking PRMT5-driven methylation and degradation of CRAF (**Figure 4**) [81]. These studies suggest that PRMT5 inhibition increases oncogenic signaling - via amplifying RAS/ERK signaling. The cellular context, tissue dependence and genetic background that explain the oncogenic vs. tumor suppressor functions of PRMT5 require further studies.

By contrast, PDGFR (platelet derived growth factor receptor) signaling in oligodendrocyte progenitor cells is positively regulated by PRMT5-methylation [82]. PRMT5-methylation of PDGFRα conceals the docking site for Cbl E3 ligase, which in turn increases PDGFRα availability and downstream activation of AKT/ERK signaling pathways required for proliferation [82].

NF-κB plays a crucial role tumorigenesis and is modified by PRMT5 in response to diverse cellular signals. As discussed above, PRMT5 activates NF-κB by direct methylation of the p65 subunit [45]. PRMT5 also interacts with the TRAIL receptor and confers resistance to TRAIL-induced apoptosis by increasing TRAIL-induced NF-κB activation, albeit, in a methyltransferase activity-independent manner [83]. PRMT5 inhibition blocks growth of multiple myeloma cells by abrogating NF-κB signaling. Mechanistically, PRMT5 methylation of TRIM21, an E3 ligase, inhibits TRIM21-dependent monoubiquitination and degradation of IKKβ through selective autophagy [84].

TGFβ signaling can both up- or down-regulate target genes involved in growth and the epithelial-mesenchymal-transition. SKI, a SMAD-interacting transcriptional repressor, is methylated by the PRMT5-MEP50-SHARPIN complex, which fine-tunes transcriptional regulation of TGFβ signaling on a specific subset of genes [53].

### Control of the DNA damage response

**Figure 5:**
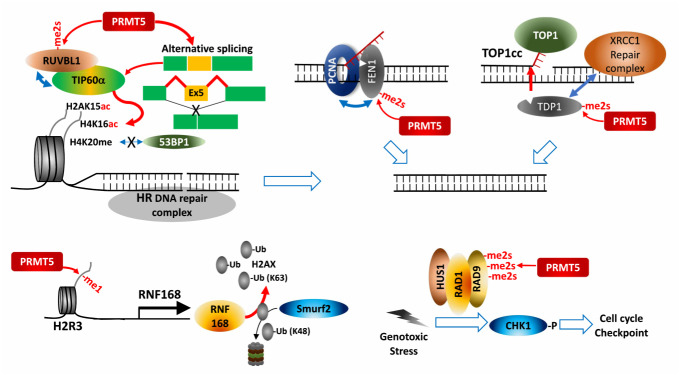
FIGURE 5: PRMT5 control of cell cycle and DNA repair. PRMT5 modify factors controlling cell cycle, thereby facilitating cell cycle checkpoint activation and homologous recombination-based DNA break repair.

The DDR plays crucial roles in maintaining genomic stability by activating cell cycle checkpoints and DNA repair processes in response to anti-cancer therapies, including ionizing radiation and chemotherapy [85–87]. Protein methylation by PRMTs is among numerous post-translational modifications that control assembly and disassembly of DDR machinery as well as related gene expression (**Figure 5**).

PRMT5 regulates expression of cell cycle/apoptosis-associated genes by modifying p53, E2F-1 and KLF4 during the DDR. PRMT5 controls H2AX protein availability by transcriptional activation of RNF168, an E3 ligase introducing K-63 ubiquitination on N-terminal K13, thereby protecting H2AX from Smurf2-mediated K-48 degradative ubiquitination on C-terminal K119. Consequently, PRMT5 ensures formation of phosphorylated H2AX (γH2AX) foci at DNA double strand break (DSB) for recruiting DNA repair proteins [88].

PRMT5 also directly modifies and controls components of DDR-associated pathways. For maturation of Okazaki fragments during replication, FEN1, a special DNA structure-specific flap endonuclease, which is important for DNA replication and repair, is recruited to replication foci by binding to PCNA (proliferating cell nuclear antigen). PRMT5 methylation of FEN1 at R192 facilitates its interaction with PCNA by inhibiting FEN1 phosphorylation at S187 by CDK1/cyclin A or CDK2/cyclin E, ensuring interaction between FEN1 and PCNA for intact DNA replication and DNA repair processes at replication and repair foci [89]. Rad9, a component of Rad9-Rad1-Hus1 complex that plays a key role in S/M and G2/M cell cycle checkpoints in response to the DDR, is modified by PRMT5, a modification that activates cell cycle checkpoints by activation of the effector kinase CHK1 [90]. PRMT5 also functions in HR-mediated DSB repair. RUVBL1, an AAA+ ATPase found in the TIP60 complex, interacts with and is methylated by PRMT5, facilitating effective demobilization of the DNA-dissection inhibitor, 53BP1, from the DSB site. As a result, TIP60-mediated H4K16 acetylation disrupts binding of 53BP1 to H4K20me2 [91]. Alternative splicing dependent TIP60 activation may also underlie PRMT5 control of HR DNA repair in hematopoietic cells [69].

Top1 (DNA topoisomerase 1) relieves DNA supercoiling during replication and transcription by forming a transient and reversible protein-ssDNA-linked structure, Top1cc (Top1 cleavage complex), that is hydrolyzed by TDP1 (tyrosyl-DNA phosphodiesterase 1). Unresolved Top1cc complexes often cause DNA DSBs and the DDR upon replication or transcription. PRMT5-catalyzed methylation of two arginines in TDP1 directly enhances TDP1 catalytic activity and promotes formation of repair foci containing the XRCC-1 complex at Top1cc-induced DNA damaged sites by facilitating interaction with XRCC1, and thereby resolving trapped Top1cc [92].

### PRMT5 in tumor immunity

Growing evidence points to a link between genetic and epigenetic alterations in oncogenesis and anti-tumor immune responses. PRMT5 depletion antagonizes melanoma growth in immunocompetent but not immunocompromised mice whereas PRMT5 overexpression accelerates tumor growth. Increased abundance of infiltrated immune cells seen upon PRMT5 inhibition coincides with enhanced anti-tumor immunity [93]. PRMT5 contributes to anti-tumor immunity by altering two distinct tumor intrinsic pathways. First, PRMT5 methylation of IFI16/IFI204, a DNA sensor that triggers IFN-I (type I interferon) responses through STING activation, suppresses STING activation and IFN-I/chemokine expression following stimuli (i.e. dsDNA; [93]). In parallel, PRMT5 was found to repress the expression of NLRC5, a master regulator of inflammasomes and antigen presentation pathways, including MHCI genes. Through the control of these complimentary pathways PRMT5 limits immune cell recruitment and activation as well as tumor recognition, which defines tumor immune evasion. In as much, PRMT5 inhibition is expected to enhance the response of cold (unresponsive) tumors to immune checkpoint therapy (ICT). Indeed, genetic or pharmacologic (GSK3326595) inhibition of PRMT5 sensitizes ICT-unresponsive cold melanoma (B16F10, or YUMM1.7) to anti-PD1 therapy [93].

Notably, PRMT5 plays several roles in hematopoiesis as in the development, activation and differentiation of diverse immune cells. Conditional PRMT5 knockout in hematopoietic cells causes bone marrow (BM) aplasia, due to loss of hematopoietic progenitor cells that exhibit severe defects in cytokine signaling [94]. Proteomic analysis of arginine-methylated peptides in human T cells identified arginine-methylated components of the TCR (T cell receptor) and transcription factors regulating T cell activation and differentiation [95]. TCR-induced PRMT5 [96] contributes to T cell maintenance (proliferation, survival and differentiation) by inducing IL-2 production [97, 98]. PRMT5 facilitates cytokine signaling through intact splicing of common gamma chain (γc)/IL2RG and JAK3 genes [99, 100]. Further, PRMT5 promotes Th17 differentiation by activation of SREBP1-choresterol pathway along the RORγt axis [101]. PRMT5 induction and subsequent Th1 and Th17 differentiation exemplify the pathological roles played by PRMT5 in autoimmune disorders, experimental autoimmune encephalomyelitis (EAE) and acute graft-versus-host diseases (aGVHD) [98, 101, 102].

Paradoxically, PRMT5 is also required for the suppressive function of regulatory T cells (Treg), which is essential to suppress autoimmunity. Mice with conditional PRMT5 knockout in Treg cells develop severe scurfy-like autoimmunity. Treg cells lacking PRMT5 or treated with PRMT5 inhibitors show loss of immunosuppressive function due to the loss of R51 methylation in FOXP3, a master regulator of Treg [103]. Notably, combination treatment of CD26Her2 mouse tumors with targeted therapy (anti-erbB2/neu antibody) plus a PRMT5 inhibitor (DS-437) enhanced anti-tumor effects through Treg suppression and augmented anti-tumor immunity [103]. Moreover, inhibitor screening identified PRMT1 methylation of FOXP3 as required for suppressive Treg function [104]. Further studies are needed define how systemic inhibition of PRMT5 will affect not only tumor cells, but various cell types found in the tumor microenvironment, including immune and other stromal cells.

## REGULATION OF PRMT5 EXPRESSION AND ACTIVITY IN CANCER

**Figure 6:**
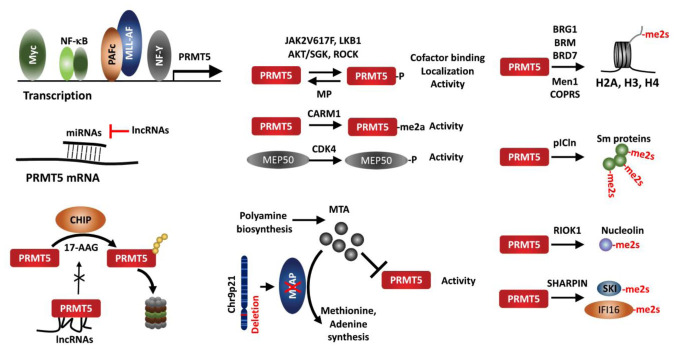
FIGURE 6: The pathways regulating PRMT5 in cancer. The activity, localization and protein-protein interaction are altered by indicated upstream components, posttranslational modification of PRMT5 or its adaptor protein, MEP50. PRMT5 interaction with adaptors serves to identify the substrates which will be methylated. PRMT5 activity is inhibited by the endogenous inhibitor, MTA, an MTAP substrate which is deleted in up to 30% of tumors.

PRMT5 expression is upregulated in most cancer types, and in most cases such upregulation is associated with poor patient survival. Given that genetic alterations (amplification, mutation, deletion) are rare in PRMT5 genes, epigenetic control of PRMT5 in cancer emerges as a key player in the control of its expression and activity (**Figure 6**).

### Transcriptional activation of PRMT5

Several transcription factors reportedly regulate PRMT5 expression. Analysis of the proximal PRMT5 promoter identified CAATT boxes that are recognized by the transcription factor NF-Y and can induce PRMT5 transcription in prostate and lung cancer lines [105]. Myc and NF-κB also upregulate PRMT5 in Eu-myc B cells, GCB (germinal center B cell-like) DLBCL (diffuse large B-cell lymphoma) tumors, and ABC (activated B cell-like) tumors [68, 106]. In AML harboring the MLL1 translocation, the polymerase-associated factor complex (PAFc), an epigenetic co-activator complex that contacts MLL1-fusion protein, is directly recruited to the PRMT5 promoter to induce PRMT5 expression [107].

### Regulation by microRNA

micro RNAs also regulate PRMT5 expression in cancer cells. In transformed B cells, miR-19a, -25, -32, -92b, and -96 negatively regulate PRMT5 expression [18]. Among them, miR-92b and -96 bind to the PRMT5 3′UTR (untranslated region) to block translation [108]. PRMT5 and miR-96 repress expression of each other via a negative feedback loop [109]. miR-1266 in PC and miR-16 and miR-4518 in glioma have been suggested to negatively regulate PRMT5 expression [110–112]. Of those, miR-16 and miR-4518 regulation of PRMT5 in glioma can be outcompeted by the long noncoding RNA (lncRNAs) LINC00015 and SNHG16, respectively [110, 112].

### Regulation by the UPS (ubiquitin-proteasome system)

The protein CHIP (carboxyl terminus of heat shock cognate 70-interacting protein), a chaperone-dependent E3 ligase functioning in protein-folding-associated degradation, reportedly promotes the proteasomal degradation of PRMT5 following treatment with the HSP90 inhibitor 17-AAG [113]. LINC01138, a lncRNA encoded in a genomic region frequently amplified in HCC, binds to and stabilizes PRMT5 protein by interfering with CHIP binding and ubiquitination [114]. Binding of PRMT5 to SHARPIN, a component of the LUBAC complex, implies a possible crosstalk between PRMT5-mediated methylation and LUBAC-driven linear ubiquitination [40, 53].

### Regulation by PTM

PRMT5 activity is itself regulated by multiple PTMs including phosphorylation/dephosphorylation, methylation, acetylation and protein-protein interactions. The JAK2 mutation JAKV617F, found in most non-CML MPN (myeloproliferative neoplasia), facilitates JAK2 interaction with and phosphorylation of PRMT5, which attenuates methylase activity by preventing interaction with MEP50 [115]. A threonine residue within the PRMT5 C-terminal tail is phosphorylated by Akt/SGK, an activity that serves as a switch to control PRMT5 targeting to the plasma membrane via choice of differential interacting partners, a PDZ domain protein or 14-3-3, depending on phosphorylation status [116]. ROCK (RhoA-activated kinase) and MP (myosin phosphatase), respectively, phosphorylate and dephosphorylate PRMT5 threonine 80, to modulate PRMT5 activity, thereby pointing to a tumor suppressor role of MP in HCC [117]. LKB1, a kinase with tumor suppressor function, phosphorylates multiple threonines (T132, 139 and 144) in the PRMT5 TIM-barrel domains required for MEP50, pICln and RIOK1 interaction, suppressing PRMT5 enzymatic activity [118]. PRMT5 itself is arginine-methylated by CARM1 (PRMT4) in the erythroleukemia cells Lys-562, which is essential for PRMT5 methyltransferase activity to repress human γ-globin expression via H4R3me2s [119]. Moreover, PRMT5 adaptors are subject to modifications that impact overall PRMT5 activity. For example, MEP50/WDR77 phosphorylation by CDK4 increases PRMT5 activity via an undefined mechanism [67]. MEP50/WDR77 is deacetylated by SIRT7 in HCT116 cells, which interfere with PRMT5/WDR77 interaction and repress PRMT5 activity [120]. Whether the impaired SIRT7/WDR77 activity and HCT116 growth are PRMT5 dependent, remains to be established.

### Regulation by protein-protein interaction (adaptors)

PRMT5 recruitment to a given substrate resulting in its methylation depends on its associated adaptor proteins, which define its conformation, activity and subcellular localization. PRMT5 interaction with MEP50/WDR77 in hetero-octameric complex increases its stability and activity [11]. PRMT5 modification of histone tails require PRMT5 interaction with BRG1, hBRM, BRD7, components of the chromatin remodeling complex [16, 17, 19, 26] or with the transcription repressor (Men1) [20, 21]. COPR5 interacts with PRMT5 and N-terminus of H4, facilitating H4R3 methylation [121]. Likewise, pICln is required for PRMT5 methylation of Sm proteins [122–124], RIOK1 for PRMT5 methylation of nucleolin [124], and SHARPIN for PRMT5 methylation of SKI and IFI16/IFI204 [53, 93]. Thus, alteration of adaptor proteins is expected to modulate PRMT5 activity and function, exemplified in MEP50 phosphorylation/acetylation [67, 120], Men1 mutation [20], and SHARPIN expression [53].

### Regulation by endogenous inhibitors

PRMT5 activity is also limited by the endogenous inhibitor, 5′methylthioadenosine (MTA), a byproduct of the polyamine synthesis pathway, which is metabolized by methylthioadenosine phosphorylase (MTAP). However, due to proximity to CDKN2A on Chr9p21, MTAP is co-deleted with CDKN2A in 15-40% of human cancers, resulting in accumulation of unmetabolized MTA and attenuation of endogenous PRMT5 activity. Accordingly, MTAP loss confers vulnerability to further inhibition of PRMT5 or of type I PRMTs [75, 125–128].

## THERAPEUTIC INTERVENTIONS BASED ON PRMT5

### Strategies to target PRMT5

Given that PRMT5 participates in a wide range of physiological processes, crucial for maintenance of cancer phenotypes, perturbation of PRMT5 is expected to provide novel means to treat cancer. Along these lines, PRMT5 plays an important role in embryonic stem and tissue specific stem/precursor cells [15, 94, 129, 130], and correspondingly, PRMT5 contributes to self-renewal of cancer stem cells (CSCs). This is exemplified by the activation of FOXP1 transcription in breast cancer stem cells [38] and DVL3 transcription, an activator of Wnt/β-catenin, in CML-LSCs (leukemic stem cells) [131], which were linked with PRMT5 activity. Given the pivotal roles of CSCs in drug-resistance and relapse, PRMT5 inhibition may provide a novel therapeutic modality for cancer-initiating cells.

Significantly, PRMT5 inhibition in established tumors could disable mechanisms protecting tumor cells from DNA damaging stress brought on by radiotherapy and chemotherapy. Indeed, PRMT5 depletion/inhibition sensitizes tumor cells to drugs inducing the DDR, exemplified by cytarabine (MLL-rearranged leukemia, [132]), PARP inhibitors (AML, [69]) and camptothecin, a topoisomerase inhibitor [92].

Emerging evidence suggests that disruption of splicing fidelity by PRMT5 depletion/inhibition could have an anti-cancer effect [72, 130]. In support of this idea, the presence of a frequently occurring splicing factor (SF) mutation (SRSF2^P95H^) or treatment with an SF3B1 inhibitor renders AML cells vulnerable to PRMT5 and/or PRMT1 inhibition [14]. Likewise, combinatorial inhibition of type I and type II PRMTs resulted in an enhanced therapeutic effect by increasing aberrant spliced targets seen upon inhibition of both PRMTs [75].

The unprecedent success of ICT is hampered by limited responses seen in a fraction of patients or by development of resistance. PRMT5 activity suppresses anti-tumor immune responses by supporting tumor intrinsic mechanisms allowing immune evasion, Treg function, and repression of IFN-1 and MHCI pathways [93, 103], providing a rationale for combining PRMT5 inhibitors with ICT. However, given the complexity of the tumor microenvironment, further studies are needed to determine how systemic PRMT5 inhibition, either singly or in combination with ICT, would affect anti-tumor immunity.

Identification of novel biomarkers is important to allow stratification of patients for precision anti-cancer treatment and improve therapeutic efficacy. The presence of SF mutations and MTAP homozygous deletion represents examples of biomarkers that aid in patient stratification for targeting PRMT5 [14, 75, 125–127].

### PRMT5 inhibitors in clinical trials

The first in-class PRMT5 inhibitor, EPZ015666, was reported in 2015 [133]. EPZ015666 binds to the PRMT5 peptide binding site, which endows peptide-competitive and SAM-uncompetitive feature of compound [133]. An improved compound, GSK3326595 (EPZ015938), is currently under two clinical trials, phase I dose escalating study in solid tumors and non-Hodgkin lymphoma (NCT02783300) and phase I safety and clinical activity study in myelodysplastic syndrome and AML (NCT03614728). In these trials, GSK3326595 monotherapy as well as combination treatment with Pembrolizumab (an anti-PD1 antibody) (NCT02783300) or 5-azacytidine (NCT03614728) will be evaluated. JNJ-64619178, a SAM-competitive inhibitor, binds to SAM- and the protein substrate binding domain of PRMT5 is being tested in a phase I clinical trial (NCT03573310) against B cell non-Hodgkin lymphoma and solid tumors. A different SAM-competitive PRMT5 inhibitor, PF-06939999, is under phase I dose escalating study in advanced or metastatic solid tumors (NCT03854227). Given the essential roles PRMT5 plays in fundamental cellular processes, it would be important to monitor possible toxicity following systemic administration of PRMT5 inhibitors. As important, efforts to improve therapeutic efficacy, probably upon combination with other therapies, may allow one to reduce PRMT5 concentration and possible toxicities.

## FUTURE PERSPECTIVES/EPILOGUE

PRMT5 regulates numerous cellular pathways but exerts major effects on histone function in transcription and RNA splicing. PRMT5 directly methylates many proteins to control their subcellular localization, protein-protein interactions, stability or activity. Many of these contribute to oncogenic transformation, and thus evaluation of potential PRMT5 inhibitors is warranted. Given the importance of PRMT5 in oncogenesis, deciphering mechanisms underlying PRMT5 expression, activity as well as subcellular localization will advance the development of advanced PRMT5-related therapeutic modalities. Thus far, researchers have largely focused on PRMT5 control of tumor intrinsic changes; however, PRMT5 effects on the tumor microenvironment, and on immune cells in particular, should be assessed in future studies. Another current knowledge gap is understanding dynamic cross talk between different PRMT family members, as exemplified by recent studies showing that PRMT1 and PRMT5 modification of a single protein may regulate opposing functions. Importantly, inhibition of one PRMT member may impact activity of another, as shown for increased PRMT5 methylation upon PRMT1 inhibition. Potential interplay among family members is critical to understand when using selective inhibitors, and in some cases, may justify targeting multiple PRMT members. Lastly, PRMT5 activity is reportedly downregulated in 30% of human cancers, due to its negative regulation by MTA, which is enhanced upon MTAP-co-deletion with CDKN2A. The notion that genetic-based stratification for PRMT5 inhibition can help map tumors vulnerable to PRMT5 targeting remains attractive and deserves to be further assessed.

## Abbreviatons:

aDMA: – asymmetric dimethylarginine;
AML: – acute myeloid leukemia;
AR: – androgen receptor;
CSC: – cancer stem cell;
DDR: – DNA damage response;
DSB: – double strand break;
HCC: – hepatocellular carcinoma;
HR: – homologous recombination;
ICT: – immune checkpoint therapy;
KO: – knockout;
lncRNA: – long noncoding RNA;
me1: – monomethylation;
m2a: – asymmetric demethylation;
m2s: – symmetric demethylation;
MMA: – monomethylarginine;
MTA: – methylthioadenosine;
MTAP: – MTA phosphorylase;
PC: – prostate cancer;
PDGF: – platelet derived growth factor;
PHD: – plant homeodomain;
PRMT: – protein arginine methyltransferase;
PTM: – posttranslational modification;
SAM: – S-adenosylmethionine;
sDMA: – symmetric dimethylarginine;
snRNP: – small nuclear ribonucleoprotein particle;
TCR: – T cell receptor;
Treg: – regulatory T cell.
